# The co-morbidity of DSM-V Gambling with DSM-V mental disorders and substance abuse in a Kenyan context of high risk schizophrenia

**DOI:** 10.1186/s12888-023-04738-4

**Published:** 2023-04-10

**Authors:** David M. Ndetei, Victoria Mutiso, Reinpeter Momanyi, Pascalyne Nyamai, Christine Musyimi, Daniel Mamah

**Affiliations:** 1 Department of Psychiatry, University of Nairobi, Nairobi, Kenya; 2 Africa Mental Health Research and Training Foundation, Nairobi, Kenya; 3 World Psychiatric Association Collaborating Centre for Research and Training, Nairobi, Kenya; 4 Department of Psychiatry, Washington University Medical School, St. Louis, MO USA

**Keywords:** Gambling, Youth, Mental disorders, Substance use, Co-morbidity, Kenyan context

## Abstract

**Introduction:**

There is evidence that gambling disorder shares similarities with other types of addictive behavior, such as occurs in substance abuse. In addition, co-morbidity of gambling with mental disorders has been established in school-going students.

**Aim:**

This study aimed at determining the comorbidity of DSM-V gambling disorder with DSM-V mental disorders and substance abuse in high school, college and university students in Kenya.

**Methods:**

This was a cross-sectional study among 536 high school, college and university students. We collected data on socio-demographic characteristics, economic indicators, DSM-V diagnosis including DSM-V gambling disorder and substance use disorders using the WHO ASSIST tool. Descriptive and inferential analyses were done.

**Results:**

A total of 536 students participated in the study, of which 11.4% (61 out of 536) had DSM-V gambling disorder. Male gender (AOR = 12.0, 95% CI: 4.99–34.3), antisocial personality disorder (AOR = 3.42, 95% CI: 1.34–8.54), tobacco use (AOR = 4.42, 95% CI: 1.15–18.3) and conduct disorder (AOR = 7.56, 95% CI: 2.34–25.1) were predictors of gambling disorder.

**Conclusion:**

Gambling is highly prevalent in Kenya learning institutions at 11.4% and is associated with mental disorders and substance use. There is a need for public awareness of gambling among Kenyan youths.

**Supplementary Information:**

The online version contains supplementary material available at 10.1186/s12888-023-04738-4.

## Background

The DSM-IV pathological gambling disorder has been re-classified as an addictive disorder in DSM V based on evidence that it has addictive characteristics and shares similarities with other types of addictive behavior, such as occurs in substance abuse [1].

The prevalence of pathological gambling in population studies in High Income Countries (HICs) ranges from 0.4% to 2.0% in the USA and Canada [2], 5.7% lifetime gambling in Sweden [3] and 30% lifetime gambling in a USA based school study [4]. High prevalence rates have been reported in South Africa, Nigeria and Ethiopia ranging between 3.2% and 28.3% in the 12–28 age group [5]. A more recent report has suggested that 54% of the youth in sub-Sahara Africa gambled, [6, 7], with the highest lifetime gambling at 76% being reported in Kenya [6, 7]. Up to 78% of University students are engaged in gambling which is more than the general population [8]. On co-morbidity, Kessler et al. 2008 reported co-morbidity of gambling with mental disorders mainly depression, while a Swedish study reported co-morbidity with cigarette and marijuana in school going students [4]. Media reports have suggested that gambling is related to suicide in Kenya, Uganda and Tanzania [9]. The significance of gambling even on a global scale is underlined by the involving access to online gambling fuelled by the aggressive marketing of betting companies [9].

An important shortcoming of these Kenya and most reports from Africa are that they are not based on clear diagnostic criteria of associated mental disorders and substance use and clearly defined socio-demographic factors and economic indicators. The objective of this study is to fill the above gaps in Africa in general and Kenya in particular on Kenyan youths attending school, college and university who had been identified as at high risk of developing schizophrenia [10].

The primary aims are: -

1. To determine the prevalence of DSM-V gambling disorder in the study population.
2. To determine the co-morbidity of gambling disorder with DSM-V mental disorders.
3. To determine the co-morbidity of gambling disorder with WHO’s Alcohol, Smoking, and Substance Involvement Screening Test (ASSIST) alcohol and substance use disorders.
4. To determine the predictors of gambling disorder.

Secondary aims are:

1. To determine the patterns of the various DSM-V symptoms of gambling disorder.
2. To determine the varying severity of gambling.
3. To determine if gambling disorder as an addictive disorder, co-exists with substance and alcohol dependency.

## Methods

### The study participants

This cross-sectional study was leveraged in a study of 536 students from high schools, colleges and universities who met the criteria of being at risk for schizophrenia using the Washington Early Recognition Center Affectivity and Psychosis (WERCAP). The students had been identified from 9,742 high school, college and university students. The details of this student population and how they were recruited have been described in several publications [11, 12].

Permission was sought from institutional heads for the tertiary academic institutions, and from the local community administration for high school participants. Inclusion criteria were the ability to read, write and speak English and informed and signed voluntary assent and consent forms. Informed consent was obtained for those over 18 years. For those under 18 years, we obtained consent from the parents/guardians and also assent from the students.

### Tools and measurements

#### Socio-demographic characteristics

A researcher-designed socio-demographic questionnaire was used to capture age, gender, the highest level of education, marital status and birth order.

#### Economic indicators tool

It records household items that are used to estimate economic status by the creation of a wealth index [13]. The wealth index used is based on the World Bank recommendation for Low Middle-Income Countries (LMICs) [13] and has been adopted by the Kenya Government for use in Kenya. It is classified into five sections; quintiles 1–5 with quintile 1 representing the lowest level of wealth and 5 the highest level.

#### The Diagnostic Interview Schedule (DIS) tool for DSM-V diagnosis

The DIS is an online tool that has questions that are read to research participants by a trained research assistant who scores the responses and an algorithm that generates DSM-V diagnoses [14]. Even as we chose this tool, which have been extensively used, we were aware of the dialogue on the validity. On the one hand, it has been argued that tools such as the DIS, may provide statistical over- estimates because they leave out important information about clients' personal history, personality style, and other contextual variables while on the other hand there is evidence that it has good diagnostic value [12, 15].

#### Drug and substance

The WHO’s ASSIST [16] was used only to determine the prevalence of different types of substance use on a ‘Yes’ or ‘No’ dimension. The WHO ASSIST has been used in Kenya [17] and other countries in Africa [18].

#### Training on data collection

This was done by one of us (DM from Washington University, St. Louis) on a face to face residential training in Kenya assisted by four researchers (a psychiatrist, public health physician, clinical psychologist and graduate nurse), who were trained on the administration of the DIS at the University of Florida by the developers of the DIS. These acted as trainers of trainees of the research assistants in Kenya.

Data collection was overseen by DMN & VM from Africa Mental Health Research and Training Foundation (AMHRTF). The lead research assistant was a university Master Level graduate nurse who was responsible for the supervision of all sites and all data collection and transmission to AMHRTF headquarters. He supervised informed consent and sat through all data collection sessions.

### Data management and analysis

Data were checked, cleaned and exported to Statistical Package for the Social Sciences (SPSS) for analysis. All data analyses were performed with SPSS version 21 (IBM, Chicago, IL). Basic descriptive statistics presented in the form of frequency and percentages were done. Chi-square tests were used for categorical data to analyze the difference in gambling disorder proportion across socio-demographics, mental disorders and substance use variables. The strength and significance of the association between the variables and gambling disorder was assessed with a 95% confidence level. Variables with a p-value less than 0.05 were then fitted into logistic regression models to identify the predictors of gambling disorder. Multivariate binomial logistic regression was employed to identify the determinants while univariate ordinal logistic regression was employed to identify the association between suicidality and severity of gambling disorder. Correlation analysis was also carried out between gambling disorder and mental disorders and substance use. Statistical significance was considered at a *p*-value < 0.05.

### Ethics approval

Ethical approval was granted by the Maseno University Ethics Review Board in Kenya (IRB number MSU/DRPI/MUERC/00344/16).

## Results

### Preamble

This being the first study of its kind, we presented as much data as possible through tables and figures for the readers and for purposes of data sharing.

### Severity of gambling disorder and prevalence of the symptoms

**Fig. 1:**
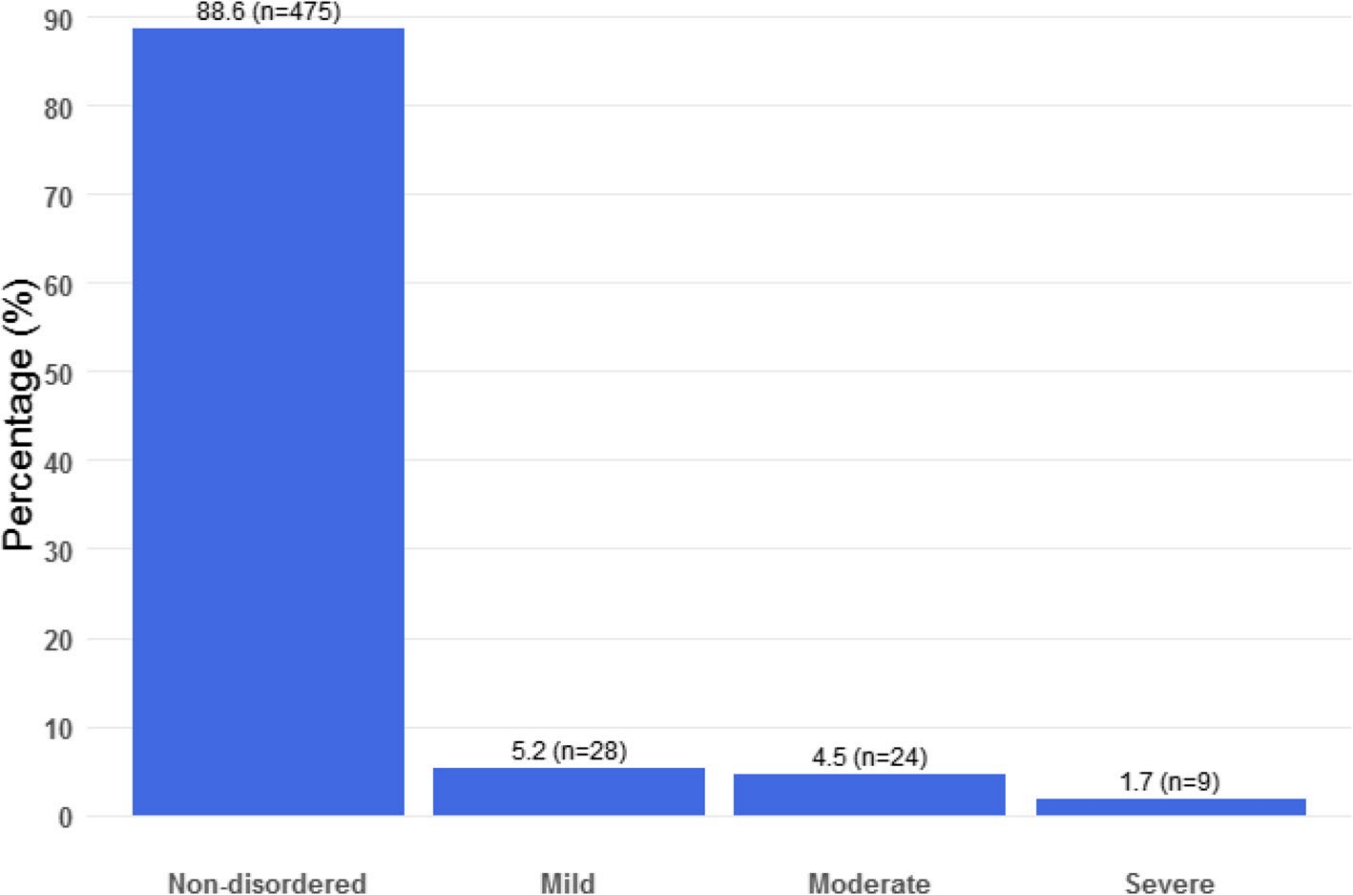
Severity of gambling disorder plot in descending order. Mild gambling disorder comprised 5.2% while moderate gambling disorder comprised 4.5% of the sample and severe gambling disorder constituted 1.7%. Non-disordered gamblers comprised of 88.6%

**Fig. 2:**
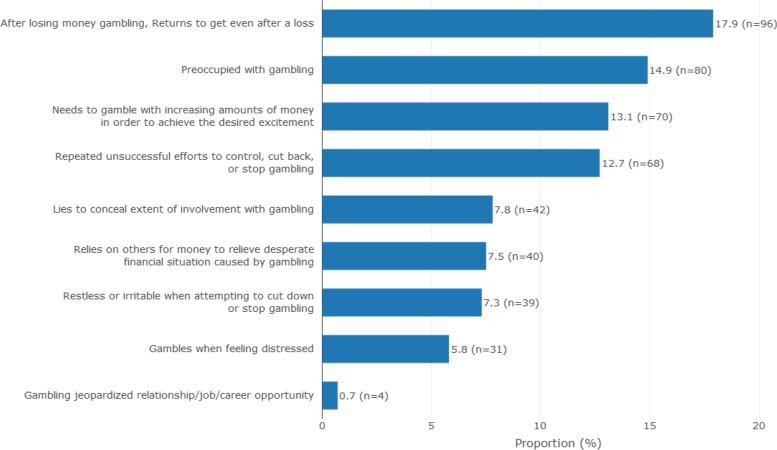
Prevalence of gambling disorder symptoms plot arranged in descending order. Most (17.9%) of the respondents gambled even after a loss with 14.9% preoccupied with gambling. Further, 13.1% of the respondents indicated to needing to gamble with more money to achieve desired excitement. In addition, 12.7% had made repeated unsuccessful attempts to reduce/stop their gambling and 7.5% of respondents had borrowed money as a result of their gambling habit

Out of 536 study participants, 11.4% (61 out of 536) had DSM V gambling disorder. There was a decreasing severity of the gambling disorder (5.2%, 4.6% and 1.7%) from mild, moderate to severe and also decreasing frequency of different types of gambling symptoms. Non-disordered gamblers comprised of 88.6% (Figs. 1 and 2).

### Gambling and socio-demographics

Of the socio-demographic variables studied, male gender was the only significant factor associated with gambling (< 0.05) as summarized in Table 1. The other nine variables were not significantly associated with gambling.

**Table 1:** Gambling and socio-demographics

Variable	Category	Total *N* = 536	Gambling disorder*		χ2	d.f	*p*-value
			**No (*N* = 475)**	**Yes (*N* = 61)**			
Age	15–19	136 (25.4%)	121 (89%)	15 (11%)	0.02	1	0.881
	20–25	400 (74.6%)	354 (88.5%)	46 (11.5%)			
Gender	Female	261 (48.7%)	255 (97.7%)	6 (2.3%)	41.60	1	**0.001**
	Male	275 (51.3%)	220 (80%)	55 (20%)			
Marital Status	Married	27 (5.05%)	26 (96.3%)	1 (3.7%)	1.81	2	0.405
	Single	507 (94.8%)	447 (88.2%)	60 (11.8%)			
	Others	1 (0.19%)	1 (100%)	0 (0%)			
Religion	Protestant	307 (57.7%)	273 (88.9%)	34 (11.1%)	0.50	3	0.919
	Catholic	184 (34.6%)	163 (88.6%)	21 (11.4%)			
	Muslim	19 (3.57%)	16 (84.2%)	3 (15.8%)			
	Other	22 (4.14%)	19 (86.4%)	3 (13.6%)			
Level of Education	High School	61 (11.4%)	53 (86.9%)	8 (13.1%)	0.21	2	0.901
	College	96 (17.9%)	85 (88.5%)	11 (11.5%)			
	University	378 (70.7%)	336 (88.9%)	42 (11.1%)			
Employment status self	Self Employed	22 (4.16%)	18 (81.8%)	4 (18.2%)	1.15	3	0.766
	Employed	8 (1.51%)	7 (87.5%)	1 (12.5%)			
	Unemployed	55 (10.4%)	48 (87.3%)	7 (12.7%)			
	Student	444 (83.9%)	395 (89%)	49 (11%)			
Employment status Mother	Self-Employed	209 (42.0%)	191 (91.4%)	18 (8.6%)	3.66	2	0.161
	Employed	124 (24.9%)	107 (86.3%)	17 (13.7%)			
	Unemployed	165 (33.1%)	141 (85.5%)	24 (14.5%)			
Employment status Father	Self-Employed	172 (39.4%)	157 (91.3%)	15 (8.7%)	3.34	2	0.188
	Employed	191 (43.8%)	166 (86.9%)	25 (13.1%)			
	Unemployed	73 (16.7%)	61 (83.6%)	12 (16.4%)			
Wealth index	Quintile 1	107 (20.0%)	97 (90.7%)	10 (9.3%)	7.59	4	0.108
	Quintile 2	99 (18.5%)	93 (93.9%)	6 (6.1%)			
	Quintile 3	107 (20.0%)	95 (88.8%)	12 (11.2%)			
	Quintile 4	107 (20.0%)	88 (82.2%)	19 (17.8%)			
	Quintile 5	115 (21.5%)	101 (87.8%)	14 (12.2%)			
Living status	Both Parents	424 (82.7%)	378 (89.2%)	46 (10.8%)	2.14	2	0.344
	Single Parent	70 (13.6%)	59 (84.3%)	11 (15.7%)			
	Other	19 (3.70%)	18 (94.7%)	1 (5.3%)			

^*^ = row percentages; χ^2^ = chi-square test value; d.f = degree of freedom; *p*-value = significance level

### Gambling and mental disorders

The DSM-V diagnoses associated (*p* < 0.05) with gambling included Obsessive–Compulsive Disorder (OCD), psychosis, agoraphobia, social phobia, drug abuse and alcohol dependence, antisocial personality disorder, conduct and oppositional defiant disorders. The WERCAP schizophrenia was also associated with gambling (Table 2).

**Table 2:** Gambling and mental disorders

Variable	Category	Total *N* = 536	Gambling disorder*		χ2	d.f	*p*-value
			**No (*N* = 475)**	**Yes (*N* = 61)**			
Major Depressive Disorder	No	358 (66.8%)	323 (90.2%)	35 (9.8%)	2.75	1	0.097
	Yes	178 (33.2%)	152 (85.4%)	26 (14.6%)			
PTSD	No	336 (62.7%)	304 (90.5%)	32 (9.5%)	3.08	1	0.079
	Yes	200 (37.3%)	171 (85.5%)	29 (14.5%)			
Bulimia/Binge Eating Disorder	No	495 (92.4%)	440 (88.9%)	55 (11.1%)	0.47	1	0.495
	Yes	41 (7.65%)	35 (85.4%)	6 (14.6%)			
Obsessive Compulsive Disorder	No	175 (32.6%)	165 (94.3%)	10 (5.7%)	8.27	1	**0.004**
	Yes	361 (67.4%)	310 (85.9%)	51 (14.1%)			
Panic Disorder	No	378 (70.5%)	340 (89.9%)	38 (10.1%)	2.24	1	0.134
	Yes	158 (29.5%)	135 (85.4%)	23 (14.6%)			
Psychosis	No	262 (48.9%)	243 (92.7%)	19 (7.3%)	8.66	1	**0.003**
	Yes	274 (51.1%)	232 (84.7%)	42 (15.3%)			
Agoraphobia	No	305 (57.0%)	286 (93.8%)	19 (6.2%)	17.71	1	** < 0.001**
	Yes	230 (43.0%)	189 (82.2%)	41 (17.8%)			
Social Phobia	No	229 (42.7%)	212 (92.6%)	17 (7.4%)	6.21	1	**0.013**
	Yes	307 (57.3%)	263 (85.7%)	44 (14.3%)			
Alcohol Abuse/Dependence	No	388 (72.4%)	353 (91%)	35 (9%)	7.76	1	**0.005**
	Yes	148 (27.6%)	122 (82.4%)	26 (17.6%)			
Drug Abuse/Dependence	No	407 (75.9%)	369 (90.7%)	38 (9.3%)	7.01	1	**0.008**
	Yes	129 (24.1%)	106 (82.2%)	23 (17.8%)			
Generalized Anxiety Disorder	No	419 (78.2%)	374 (89.3%)	45 (10.7%)	0.78	1	0.377
	Yes	117 (21.8%)	101 (86.3%)	16 (13.7%)			
Somatization Disorder	No	364 (67.9%)	324 (89%)	40 (11%)	0.17	1	0.678
	Yes	172 (32.1%)	151 (87.8%)	21 (12.2%)			
Hypochondriasis	No	340 (63.4%)	306 (90%)	34 (10%)	1.76	1	0.185
	Yes	196 (36.6%)	169 (86.2%)	27 (13.8%)			
ASQ Autism	No Risk	441 (82.3%)	395 (89.6%)	46 (10.4%)	5.29	2	0.071
	Low Risk	90 (16.8%)	77 (85.6%)	13 (14.4%)			
	High Risk	5 (0.93%)	3 (60%)	2 (40%)			
Wercap Schizophrenia	Low Risk	263 (49.1%)	247 (93.9%)	16 (6.1%)	14.37	2	** < 0.001**
	Moderate Risk	6 (1.12%)	5 (83.3%)	1 (16.7%)			
	High Risk	267 (49.8%)	223 (83.5%)	44 (16.5%)			
Attention deficit/hyperactivity disorder	Negative	533 (99.4%)	472 (88.6%)	61(11.4%)	0.39	1	0.534
	Criteria met for ADHD, Predominately hyperactive/impulsive presentation	3 (0.56%)	3 (100%)	0 (0%)			
Antisocial personality disorder	Negative	460 (85.8%)	417 (90.7%)	43 (9.3%)	32.80	2	** < 0.001**
	All criteria met except possible exclusion	52 (9.70%)	34 (65.4%)	18 (34.6%)			
	Indeterminate	24 (4.48%)	24 (100%)	0 (0%)			
Conduct disorder	Negative	458 (85.4%)	420 (91.7%)	38 (8.3%)	48.16	2	** < 0.001**
	All criteria met except exclusion for Antisocial Personality	25 (4.66%)	12 (48%)	13 (52%)			
	Indeterminate	53 (9.89%)	43 (81.1%)	10 (18.9%)			
Oppositional defiant disorder	Negative	520 (97.0%)	464 (89.2%)	56 (10.8%)	6.54	2	**0.038**
	All criteria met except exclusion	9 (1.68%)	6 (66.7%)	3 (33.3%)			
	Indeterminate	7 (1.31%)	5 (71.4%)	2 (28.6%)			

* = row percentages; χ^2^ = chi-square test value; d.f = degree of freedom; *p*-value = significance level

### Gambling and substance use

Gambling was associated (*p* < 0.05) with all substances lifetime use except for amphetamine lifetime and inhalants lifetime use and with all substances current use except for amphetamine, hallucinogens and khat (a psych-stimulant herb) (Table 3).

**Table 3:** Gambling and substance use (Lifetime and Current)

Variable	Category	Total *N* = 536	Gambling disorder*		χ2	d.f	*p*-value
			**No (*N* = 475)**	**Yes (*N* = 61)**			
**Lifetime**							
Tobacco lifetime	No	480 (89.6%)	434 (90.4%)	46 (9.6%)	14.72	1	** < 0.001**
	Yes	56 (10.4%)	41 (73.2%)	15 (26.8%)			
Alcohol lifetime	No	406 (75.7%)	366 (90.1%)	40 (9.9%)	3.88	1	**0.049**
	Yes	130 (24.3%)	109 (83.8%)	21 (16.2%)			
Cannabis lifetime	No	491 (92.1%)	440 (89.6%)	51 (10.4%)	6.88	1	**0.009**
	Yes	42 (7.88%)	32 (76.2%)	10 (23.8%)			
Cocaine lifetime	No	524 (98.1%)	468 (89.3%)	56 (10.7%)	14.99	1	** < 0.001**
	Yes	10 (1.87%)	5 (50%)	5 (50%)			
Amphetamine lifetime	No	523 (97.8%)	465 (88.9%)	58 (11.1%)	2.25	1	0.134
	Yes	12 (2.24%)	9 (75%)	3 (25%)			
Inhalants lifetime	No	528 (98.7%)	469 (88.8%)	59 (11.2%)	2.07	1	0.150
	Yes	7 (1.31%)	5 (71.4%)	2 (28.6%)			
Sedatives lifetime	No	513 (95.9%)	458 (89.3%)	55 (10.7%)	5.72	1	**0.017**
	Yes	22 (4.11%)	16 (72.7%)	6 (27.3%)			
Hallucinogens lifetime	No	526 (98.3%)	468 (89%)	58 (11%)	4.36	1	**0.037**
	Yes	9 (1.68%)	6 (66.7%)	3 (33.3%)			
Opioids lifetime	No	524 (97.9%)	467 (89.1%)	57 (10.9%)	6.93	1	**0.008**
	Yes	11 (2.06%)	7 (63.6%)	4 (36.4%)			
Khat lifetime	No	519 (96.8%)	463 (89.2%)	56 (10.8%)	5.66	1	**0.017**
	Yes	17 (3.17%)	12 (70.6%)	5 (29.4%)			
**Current**							
Tobacco current	No	502 (93.7%)	451 (89.8%)	51 (10.2%)	11.70	1	** < 0.001**
	Yes	34 (6.34%)	24 (70.6%)	10 (29.4%)			
Alcohol current	No	429 (80.0%)	386 (90%)	43 (10%)	3.93	1	**0.048**
	Yes	107 (20.0%)	89 (83.2%)	18 (16.8%)			
Cannabis current	No	504 (94.0%)	452 (89.7%)	52 (10.3%)	9.46	1	**0.002**
	Yes	32 (5.97%)	23 (71.9%)	9 (28.1%)			
Cocaine current	No	531 (99.1%)	473 (89.1%)	58 (10.9%)	11.83	1	** < 0.001**
	Yes	5 (0.93%)	2 (40%)	3 (60%)			
Amphetamine current	No	526 (98.1%)	468 (89%)	58 (11%)	3.50	1	0.061
	Yes	10 (1.87%)	7 (70%)	3 (30%)			
Inhalants current	No	533 (99.4%)	474 (88.9%)	59 (11.1%)	9.14	1	**0.002**
	Yes	3 (0.56%)	1 (33.3%)	2 (66.7%)			
Sedatives current	No	523 (97.6%)	467 (89.3%)	56 (10.7%)	9.69	1	**0.002**
	Yes	13 (2.43%)	8 (61.5%)	5 (38.5%)			
Hallucinogens current	No	533 (99.4%)	473 (88.7%)	60 (11.3%)	1.44	1	0.230
	Yes	3 (0.56%)	2 (66.7%)	1 (33.3%)			
Opioids current	No	530 (98.9%)	472 (89.1%)	58 (10.9%)	8.97	1	**0.003**
	Yes	6 (1.12%)	3 (50%)	3 (50%)			
Khat current	No	526 (98.1%)	467 (88.8%)	59 (11.2%)	0.75	1	0.386
	Yes	10 (1.87%)	8 (80%)	2 (20%)			

^*^ = row percentages; χ^2^ = chi-square test value; d.f = degree of freedom; *p*-value = significance level

### Independent predictors

The independent predictors of gambling (*p* < 0.05) were male gender, antisocial personality behavior, tobacco lifetime use, WERCAP schizophrenia and conduct disorders (Table 4).

**Table 4:** Independent Predictors of gambling disorder

Variable	Category	Model 1	Model 2
		**AOR (95% CI)**	**AOR (95% CI)**
Gender	Female	Ref	Ref
	Male	12.0 (4.99–34.3)***	11.5 (4.87–32.3)***
Obsessive Compulsive Disorder Psychosis	No	Ref	Ref
	Yes	1.91 (0.76–5.08)	1.70 (0.69–4.45)
	No	Ref	Ref
Agoraphobia	Yes	0.52 (0.20–1.31)	0.58 (0.24–1.44)
	No	Ref	Ref
Social Phobia	Yes	1.74 (0.72–4.41)	2.03 (0.85–5.03)
	No	Ref	Ref
Alcohol Abuse/Dependence	Yes	0.89 (0.33–2.34)	0.92 (0.35–2.41)
	No	Ref	Ref
Drug Abuse/Dependence	Yes	1.20 (0.49–2.92)	1.14 (0.48–2.67)
	No	Ref	Ref
Wercap Schizophrenia	Yes	0.73 (0.30–1.74)	0.70 (0.29–1.63)
	Low Risk	Ref	Ref
	Moderate Risk	29.0 (0.75–1,417)	10.8 (0.30–402)
	High Risk	2.60 (1.01–6.87)*	2.49 (0.98–6.48)
Tobacco lifetime	No	Ref	—
	Yes	4.42 (1.15–18.3)*	—
Alcohol lifetime	No	Ref	—
	Yes	0.54 (0.16–1.50)	—
Cannabis lifetime	No	Ref	—
	Yes	0.45 (0.11–1.79)	—
Cocaine lifetime	No	Ref	—
	Yes	5.90 (0.82–44.5)	—
Sedatives lifetime	No	Ref	—
	Yes	0.27 (0.04–1.49)	—
Hallucinogens lifetime	No	Ref	—
	Yes	4.27 (0.42–41.9)	—
Opioids lifetime	No	Ref	—
	Yes	5.40 (0.76–36.1)	—
Khat lifetime	No	Ref	—
	Yes	0.33 (0.06–1.64)	—
Tobacco current	No	—	Ref
	Yes	—	1.89 (0.50–6.83)
Alcohol current	No	—	Ref
	Yes	—	0.75 (0.27–1.81)
Cannabis current	No	—	Ref
	Yes	—	0.72 (0.16–2.92)
Cocaine current	No	—	Ref
	Yes	—	2.34 (0.15–42.4)
Inhalants current	No	—	Ref
	Yes	—	1.51 (0.04–107)
Sedatives current	No	—	Ref
	Yes	—	1.08 (0.12–8.17)
Opioids current	No	—	Ref
	Yes	—	5.84 (0.66–54.7)
Antisocial personality disorder	Negative	Ref	Ref
	All criteria met except possible exclusion	3.42 (1.34–8.54)**	3.01 (1.17–7.55)*
	Indeterminate	0.00 (0.00–612,855)	0.00 (0.00–586,413)
Conduct disorder	Negative	Ref	Ref
	All criteria met except exclusion for Antisocial Personality	7.56 (2.34–25.1)***	5.51 (1.68–18.2)**
	Indeterminate	1.92 (0.77–4.51)	1.62 (0.64–3.85)
Oppositional defiant disorder	Negative	Ref	Ref
	All criteria met except exclusion	0.52 (0.05–4.54)	0.57 (0.05–4.89)
	Indeterminate	3.21 (0.19–36.0)	1.47 (0.09–20.1)

*AOR *Adjusted Odds Ratio, *CI *Confidence interval; *p < 0.05; **p < 0.01; ***p < 0.001; model 1 = multivariate logistic regression where current substance use for Tobacco, alcohol, cannabis, cocaine, inhalants, sedatives and opioids are omitted; model 2 = multivariate logistic regression where lifetime substance use for tobacco, alcohol, cannabis, cocaine, sedatives, hallucinogens, opioids and khat are omitted

### Gambling and suicidality

Table 5 summarizes the association between gambling disorder and suicide. We found significant association between severity of gambling and suicidality.

**Table 5:** Gambling and Suicidality

Variable	Category	Total	Severity Criteria for Gambling Disorder*				χ2	d.f	*p*-value
		***N* = 536**	**Non- disordered**	**Mild**	**Moderate**	**Severe**			
Suicidality	No	498(92.9%)	445(93.7%)	27(96.4%)	22(91.7%)	4(44.4%)	33.11	3	** < 0.001**
	Yes	38(7.1%)	30(6.3%)	1(3.6%)	2(8.3%)	5(55.6%)			
**Variable**	**Category**	**OR (95% CI)**^**†**^	***p*-value**^**†**^						
Suicidality	No	Ref							
	Yes	2.62 (1.13—6.05)	**0.024**						

^*^ = column percentages; † = ordinal logistic regression model; χ2 = chi-square test value; OR = Crude Odds Ratio; CI = confidence interval; d.f = degree of freedom; *p*-value = significance level

### Correlation between gambling and DSM V/ Substance use disorders

Most of the studied disorders had a significant (*p* < 0.05) correlation with gambling as summarized in supplementary Tables S1, S2 and S3.

## Discussion

As far as we were able to determine from the literature on global and African studies, we present the first study reported in Africa and Kenya in particular that examines a wide range of factors associated with gambling disorder in youth aged 15–25 and attending educational institutions (high school, college and university). We describe the prevalence of DSM-V gambling disorder, the severity of the gambling disorder, patterns of individual symptoms of gambling, associated socio-demographic and economic indicators and co-morbidity with DSM-V disorders, WHO ASSIST substance abuse disorders and suicidality (ideas and behavior). This combination of multiple study variables serves to inform a more inclusive intervention.

The prevalence of 11.4% of DSM-V gambling disorder found in this study is far less than the 54–76% reported so far in Kenya, Uganda and Africa in general and also less than the 30% reported in USA schools. The explanation for this disparity of finding is mainly on the methodology we used – i.e. clear diagnostic criteria as opposed to media reports – in Kenya. This is in spite of the fact that this study was on students at risk of developing schizophrenia, though not yet developed schizophrenia. Of this 11.4%, the gambling disorder comes in various degrees of severity. It is noteworthy that of the DSM-V symptoms of gambling disorder, the leading 4 symptoms in prevalence (Q6, Q4, Q1, Q3) in the descending order are addiction behaviors, providing Kenyan evidence in support for re-classifying gambling as an addiction disorder amongst other drug addiction disorders. Also of note is that students identified money as a driving factor (Q1, Q6, Q9) even though there was no significant association between gambling and economic indicators (employment status of the guardian/parents and the wealth index). This would suggest a directional cause from addictive gambling to money concerns to sustain the addiction. The students did not associate gambling with any lost educational opportunities or loss of social relationships with only 0.7% scoring positive for the symptoms.

Male gender was the only socio-demographic variable significantly associated with gambling. Anti-social personality disorder, conduct disorder and oppositional disorder, all significantly (*p* < 0.05) associated with gambling (Table 3) are disorders that are largely associated with the male gender and therefore not surprising that the male gender was the only socio demographic factor that was associated with gambling. It is noteworthy that there was no difference in age structure suggesting that gambling occurs equally across the ages. This is not surprising given that advertising of gambling through radio/television and social media and given the almost-universal access to mobile phones, all age groups are equally targeted. All these suggest that gambling is primarily a learned behavior through advertisement with "promises" of huge gains in money, reinforced from time to time by occasional money gains.

It is noteworthy that gambling was not associated with major depression (*p* > 0.05). The psychiatric disorders significantly (*p* < 0.05) associated with gambling were agoraphobia and social phobia which are more indicative of social disconnect rather than social connect and so are the personality disorders (anti-social, conduct and oppositional defiant), DSM-V psychosis and WERCAP schizophrenia suggesting that gambling is not a sign of shared group activity but individual driven activity.

Being an addiction disorder as per its leading scores on DSM-V symptoms, it is not surprising that gambling disorder is co-morbid with both lifetime and current use of most of the WHO-ASSIST listed drugs of abuse. The significant association of OCD and alcohol abuse/dependence and drug abuse is not unexpected, given that they share symptoms of repeated behaviors.

The two different models used to predict gambling produced similar results, confirming the validity of the predictors. These predictors include male gender, antisocial personality and conduct disorders, high risk factors for psychosis and tobacco both lifetime and current. Apart from high risk WERCAP for schizophrenia, no particular mental disorder was a predictor of gambling.

Our findings provide an evidence base for the need for (i) enhanced public awareness on the prevalence of gambling and its association with mental disorders, substance use and high levels of suicidality in Kenya learning institutions; (ii) screening for mental, alcohol and substance use and suicidality in cases of gambling; (iii) controlled advertisement of gambling and (iv) gambling to be viewed indeed as an addiction disorder.

## Conclusions

1. Gambling is highly prevalent in Kenya learning institutions at 11.4% though still less than previously reported in media reports
2. It occurs in equal prevalence in the age group 15–19 (found in schools) and 20–25 (found in college and university levels)
3. Gambling is associated with mental disorders – namely agoraphobia, social phobia, psychosis and suicidality
4. Gambling is associated with most substance of abuse
5. There is a need for public awareness, control of advertisement and access to gambling apps for the Kenyan youths
6. Gambling needs a broad-based public and clinical approach.

### Limitations and strength

This study consisted of a subset of 536 study participants who had scored positive for high risk psychosis (not yet converted), thus the high rates of DSM-V psychosis in our findings (51.1%) and the high rate of various degrees of WERCAP psychosis. However, apart from the psychosis which we have explained, our data provide information on other associations of gambling disorder that may be useful for a clinical and public awareness campaign.

As already mentioned in the methodology, there have been arguments for and against the use of DIS as a valid tool. We do not know how precisely these different arguments may have affected our results. But we lean on the strengths that we had already validated WERCAP in our socio-cultural setting.

A major strength of this study is that for all the diagnoses we used well defined DSM-V criteria for DSM-V diagnoses and WHO ASSIST for alcohol and various substance use.

## Supplementary Information

**Additional file 1: Supplementary Table S1.** Correlation between Gambling and DSM V disorders.** Supplementary Table S2.** Correlation between Gambling and lifetime substance use. **Supplementary Table S3.** Correlation between Gambling and current substance use.

## Data Availability

The datasets used and/or analyzed during the current study are available from the corresponding author on reasonable request.

## Abbreviations

AMHRTF: Africa Mental Health Research and Training Foundation
ASSIST: Alcohol, Smoking, and Substance Involvement Screening Test
DIS: Diagnostic Interview Schedule
DSM-V: Diagnostic and Statistical Manual, 5^th^ Edition
HIC: High Income Countries
LMICs: Low Middle-Income Countries
OCD: Obsessive Compulsive Disorder
WERCAP: Washington Early Recognition Center Affectivity and Psychosis
WHO: World Health Organization
