# Supplementary material for: The co-morbidity of DSM-V Gambling with DSM-V mental disorders and substance abuse in a Kenyan context of high risk schizophrenia
Source: BMC Psychiatry. 2023 Apr 10;23:239. doi: 10.1186/s12888-023-04738-4 (PMC10084586; doi:10.1186/s12888-023-04738-4)
Supplement: Supplementary file 1 — Additional file 1: Supplementary Table S1. Correlation between Gambling and DSM V disorders. Supplementary Table S2. Correlation between Gambling and lifetime substance use. Supplementary Table S3. Correlation between Gambling and current substance use. [file 12888_2023_4738_MOESM1_ESM.doc]

**Supplementary Table S1: Correlation between Gambling and DSM V disorders**

| **Pearson Correlations** | **1** | **2** | **3** | **4** | **5** | **6** | **7** | **8** | **9** | **10** | **11** | **12** | **13** | **14** | **15** | **16** | **17** | **18** | **19** | **20** | **21** |
| --- | --- | --- | --- | --- | --- | --- | --- | --- | --- | --- | --- | --- | --- | --- | --- | --- | --- | --- | --- | --- | --- |
| 1. Gambling disorder | 1 |  |  |  |  |  |  |  |  |  |  |  |  |  |  |  |  |  |  |  |  |
| 2. Major Depressive Disorder | .072 | 1 |  |  |  |  |  |  |  |  |  |  |  |  |  |  |  |  |  |  |  |
| 3. PTSD | .076 | .472** | 1 |  |  |  |  |  |  |  |  |  |  |  |  |  |  |  |  |  |  |
| 4. Bulimia/Binge Eating Disorder | .029 | .319** | .242** | 1 |  |  |  |  |  |  |  |  |  |  |  |  |  |  |  |  |  |
| 5. Obsessive Compulsive Disorder | .124** | .305** | .406** | .185** | 1 |  |  |  |  |  |  |  |  |  |  |  |  |  |  |  |  |
| 6. Panic Disorder | .065 | .465** | .432** | .353** | .398** | 1 |  |  |  |  |  |  |  |  |  |  |  |  |  |  |  |
| 7. Psychosis | .127** | .412** | .423** | .267** | .481** | .444** | 1 |  |  |  |  |  |  |  |  |  |  |  |  |  |  |
| 8. Agoraphobia | .182** | .332** | .437** | .204** | .396** | .406** | .473** | 1 |  |  |  |  |  |  |  |  |  |  |  |  |  |
| 9. Social Phobia | .108* | .385** | .440** | .163** | .444** | .360** | .468** | .568** | 1 |  |  |  |  |  |  |  |  |  |  |  |  |
| 10. Alcohol Abuse/Dependence | .120** | .309** | .300** | .183** | .314** | .287** | .320** | .303** | .348** | 1 |  |  |  |  |  |  |  |  |  |  |  |
| 11. Drug Abuse/Dependence | .114** | .270** | .270** | .166** | .262** | .277** | .306** | .296** | .292** | .609** | 1 |  |  |  |  |  |  |  |  |  |  |
| 12. Generalized Anxiety Disorder | .038 | .481** | .339** | .341** | .233** | .421** | .327** | .408** | .374** | .128** | .104* | 1 |  |  |  |  |  |  |  |  |  |
| 13. Somatization Disorder | .018 | .355** | .337** | .253** | .291** | .353** | .344** | .368** | .376** | .246** | .286** | .362** | 1 |  |  |  |  |  |  |  |  |
| 14. Hypochondriasis | .057 | .411** | .431** | .233** | .405** | .452** | .487** | .456** | .460** | .354** | .388** | .405** | .515** | 1 |  |  |  |  |  |  |  |
| 15. ASQ Autism | .080 | .084 | .119** | .023 | .122** | .124** | .216** | .174** | .144** | .216** | .253** | .046 | .086* | .154** | 1 |  |  |  |  |  |  |
| 16. Wercap Schizophrenia | .163** | .572** | .526** | .231** | .393** | .464** | .570** | .466** | .465** | .356** | .316** | .441** | .332** | .516** | .206** | 1 |  |  |  |  |  |
| 17. Attention deficit/hyperactivity disorder | -.027 | .106* | .097* | -.022 | -.001 | -.049 | .023 | .036 | .014 | .066 | -.042 | .021 | -.052 | -.057 | -.034 | .075 | 1 |  |  |  |  |
| 18. Antisocial personality disorder | .127** | .160** | .105* | .040 | .100* | .100* | .068 | .032 | .057 | .059 | .080 | .066 | .035 | .059 | -.019 | .183** | .034 | 1 |  |  |  |
| 19. Conduct disorder | .197** | .077 | .020 | .022 | .071 | .048 | .152** | .110* | .040 | .084 | .089* | .049 | .016 | .010 | .038 | .089* | -.030 | .175** | 1 |  |  |
| 20. Compulsive disorder | .060 | .124** | .083 | .047 | .053 | .087* | .123** | .039 | .045 | .015 | .056 | .066 | -.026 | .036 | .048 | .089* | -.011 | .094* | .114** | 1 |  |
| 21. Oppositional defiant disorder | .105* | .038 | .013 | .100* | .044 | .063 | .045 | .046 | .024 | .032 | .048 | .069 | .052 | .016 | .029 | .074 | -.013 | .179** | .224** | .144** | 1 |
| **= Correlation is significant at the 0.01 level (2-tailed); *= Correlation is significant at the 0.05 level (2-tailed) | | | | | | | | | | |  |  |  |  |  |  |  |  |  |  |  |

**Supplementary Table S2: Correlation between Gambling and lifetime substance use**

| **Pearson Correlations** | **1** | **2** | **3** | **4** | **5** | **6** | **7** | **8** | **9** | **10** | **11** |
| --- | --- | --- | --- | --- | --- | --- | --- | --- | --- | --- | --- |
| 1. Gambling disorder | 1 |  |  |  |  |  |  |  |  |  |  |
| 2. Tobacco lifetime | .166** | 1 |  |  |  |  |  |  |  |  |  |
| 3. Alcoholic lifetime | .085* | .561** | 1 |  |  |  |  |  |  |  |  |
| 4. Cannabis lifetime | .114** | .610** | .490** | 1 |  |  |  |  |  |  |  |
| 5. Cocaine lifetime | .168** | .229** | .246** | .219** | 1 |  |  |  |  |  |  |
| 6. Amphetamine lifetime | .065 | .157** | .151** | .143** | .165** | 1 |  |  |  |  |  |
| 7. Inhalants lifetime | .062 | .178** | .204** | .150** | .348** | .538** | 1 |  |  |  |  |
| 8. Sedatives lifetime | .103* | .302** | .279** | .184** | .458** | .477** | .390** | 1 |  |  |  |
| 9. Hallucinogens lifetime | .090* | .099* | .198** | .089* | .304** | .373** | .369** | .339** | 1 |  |  |
| 10. Opioids lifetime | .114** | .081 | .226** | .124** | .369** | .156** | .099* | .302** | .391** | 1 |  |
| 11. Khat lifetime | .103* | .286** | .245** | .264** | .211** | .260** | .354** | .177** | .142** | .124** | 1 |
| **= Correlation is significant at the 0.01 level (2-tailed); *= Correlation is significant at the 0.05 level (2-tailed) | | | | | | | | | | | |

**Supplementary Table S3: Correlation between Gambling and current substance use**

| **Pearson Correlations** | **1** | **2** | **3** | **4** | **5** | **6** | **7** | **8** | **9** | **10** | **11** |
| --- | --- | --- | --- | --- | --- | --- | --- | --- | --- | --- | --- |
| 1. Gambling disorder | 1 |  |  |  |  |  |  |  |  |  |  |
| 2. Tobacco current | .148** | 1 |  |  |  |  |  |  |  |  |  |
| 3. Alcoholic current | .086* | .464** | 1 |  |  |  |  |  |  |  |  |
| 4. Cannabis current | .133** | .419** | .445** | 1 |  |  |  |  |  |  |  |
| 5. Cocaine current | .149** | .214** | .146** | .303** | 1 |  |  |  |  |  |  |
| 6. Amphetamine current | .081 | .134** | .069 | .140** | .273** | 1 |  |  |  |  |  |
| 7. Inhalants current | .131** | .186** | .150** | .192** | .513** | .544** | 1 |  |  |  |  |
| 8. Sedatives current | .134** | .357** | .194** | .216** | .363** | .426** | .476** | 1 |  |  |  |
| 9. Hallucinogens current | .052 | -.020 | .025 | .087* | .513** | .359** | .330** | .313** | 1 |  |  |
| 10. Opioids current | .129** | .118** | .169** | .123** | .359** | .247** | .230** | .214** | .467** | 1 |  |
| 11. Khat current | .037 | .190** | .173** | .140** | .417** | .287** | .359** | .158** | .174** | .247** | 1 |
| **= Correlation is significant at the 0.01 level (2-tailed); *= Correlation is significant at the 0.05 level (2-tailed) | | | | | | | |  |  |  |  |
